# Evidence for GABA‐A receptor dysregulation in gambling disorder: correlation with impulsivity

**DOI:** 10.1111/adb.12457

**Published:** 2016-10-13

**Authors:** Inge Mick, Anna C. Ramos, Jim Myers, Paul R. Stokes, Samantha Chandrasekera, David Erritzoe, Maria A. Mendez, Roger N. Gunn, Eugenii A. Rabiner, Graham E. Searle, José C. F. Galduróz, Adam D. Waldman, Henrietta Bowden‐Jones, Luke Clark, David J. Nutt, Anne R. Lingford‐Hughes

**Affiliations:** ^1^ Centre for Neuropsychopharmacology, Division of Brain Sciences, Faculty of Medicine Imperial College London UK; ^2^ Department of Psychobiology Universidade Federal de São Paulo Brazil; ^3^ Centre for Affective Disorders, Department of Psychological Medicine Institute of Psychiatry, Psychology and Neuroscience, King's College London UK; ^4^ Forensic and Neurodevelopmental Sciences Institute of Psychiatry, King's College UK; ^5^ Imanova Ltd. Centre for Imaging Sciences UK; ^6^ Department of Neuroimaging Institute of Psychiatry, King's College UK; ^7^ Department of Imaging, Division of Experimental Medicine, Department of Medicine Imperial College UK; ^8^ National Problem Gambling Clinic, CNWL NHS Foundation Trust Imperial College London UK; ^9^ Centre for Gambling Research at UBC, Department of Psychology University of British Columbia Canada

**Keywords:** GABA system, gambling disorder, [^11^C]Ro15‐4513 PET

## Abstract

As a behavioural addiction, gambling disorder (GD) provides an opportunity to characterize addictive processes without the potentially confounding effects of chronic excessive drug and alcohol exposure. Impulsivity is an established precursor to such addictive behaviours, and GD is associated with greater impulsivity. There is also evidence of GABAergic dysregulation in substance addiction and in impulsivity. This study therefore investigated GABA_A_ receptor availability in 15 individuals with GD and 19 healthy volunteers (HV) using [^11^C]Ro15‐4513, a relatively selective α5 benzodiazepine receptor PET tracer and its relationship with impulsivity. We found significantly higher [^11^C]Ro15‐4513 total distribution volume (*V_T_*) in the right hippocampus in the GD group compared with HV. We found higher levels of the ‘Negative Urgency’ construct of impulsivity in GD, and these were positively associated with higher [^11^C]Ro15‐4513 *V_T_* in the amygdala in the GD group; no such significant correlations were evident in the HV group. These results contrast with reduced binding of GABAergic PET ligands described previously in alcohol and opiate addiction and add to growing evidence for distinctions in the neuropharmacology between substance and behavioural addictions. These results provide the first characterization of GABA_A_ receptors in GD with [^11^C]Ro15‐4513 PET and show greater α5 receptor availability and positive correlations with trait impulsivity. This GABAergic dysregulation is potential target for treatment.

## Introduction

There is considerable overlap between gambling disorder (GD) (previously termed pathological gambling) with drug and alcohol addiction with regard to clinical phenomenology and treatment, comorbidity, heritability and neurobiological profile (Clark 2014; Clark and Limbrick‐Oldfield 2013). Predicated on such evidence, this condition was recently reclassified from an ‘impulse control disorder’ in DSM‐IV to the ‘substance‐related and addictive disorders’ in DSM‐5 (Clark 2014). As a behavioural addiction, GD provides an opportunity to characterize addictive processes without the potentially confounding effects of chronic excessive drug/alcohol exposure. Compared with drug or alcohol addiction, little is known about the neuropharmacology of GD, and a better characterization of its neurobiology will inform developments in the prevention and treatment of both substance and behavioural addictions.

Recent studies using positron emission tomography (PET) to directly assess the dopamine and opioid systems in GD have demonstrated some unexpected differences to established findings in substance addictions. For instance, in contrast to studies in cocaine and alcohol addiction, [^11^C]raclopride and [^11^C]‐(+)‐PHNO PET revealed no differences in dopamine receptor DRD2/3 availability and greater stimulant‐induced dopamine release in individuals with GD compared with healthy volunteers (HV; Albein‐Urios *et al.* 2012a,2012b; Boileau *et al.* 2013; Boileau *et al.* 2014). A key modulator of the dopaminergic system is the mu opioid receptor (MOR), and opiate receptor antagonists have some efficacy in treating GD. Higher MOR availability has been reported in studies by using [^11^C]carfentanil or [^11^C]diprenorphine PET in cocaine, opiate and alcohol addiction (Gorelick *et al.* 2005; Heinz *et al.* 2005; Williams *et al.* 2007; Williams *et al.* 2009). However, we recently reported no difference in [^11^C]carfentanil binding in individuals with GD (Mick *et al.* 2015). Thus, *in vivo* PET imaging studies of neuropharmacology suggest that GD shows important differences to substance addiction.

The GABAergic system is another key modulator of mesolimbic dopaminergic processes but has received scant attention in the context of addictions (Hayes *et al.* 2014). With the [^11^C]Ro15‐4513 PET radiotracer, which is relatively selective for the α5 subtype of the benzodiazepine receptor, we observed in alcohol and in heroin addiction lower levels of limbic [^11^C]Ro15 4513 binding, particularly in the nucleus accumbens (NAc) and right hippocampus (Lingford‐Hughes *et al.* 2016; Lingford‐Hughes *et al.* 2012a). We have also shown that [^11^C]Ro15 4513 binding was higher in participants with a history of tobacco smoking (Stokes *et al.* 2013) .

With regard to gambling, Nussbaum *et al.* (2011) proposed that GABAergic modulation of opioid release in the brain reward system may be important to the ‘rush’ experienced by gamblers in response to major jackpots and that differences in this modulation could distinguish individuals with GD from non‐dependent gamblers. There is limited further evidence regarding the GABAergic system in individuals with GD, with some inconsistent evidence of greater GABA concentrations measured in the CSF in GD (Nordin and Sjodin 2007; Roy *et al.* 1989). These observations, as well as the growing use of GABAergic medication such as baclofen and topiramate to treat addiction, indicate that it is timely and important to characterize the GABA_A_ receptor system in GD (Lingford‐Hughes *et al.* 2012b).

A major feature common to gambling and substance‐related disorders is impaired impulse control, which may be fundamental to both the development and maintenance of addictive disorders (Bechara 2005; Lawrence *et al.* 2009; Verdejo‐Garcia *et al.* 2008). We have previously shown that Negative Urgency (NU) derived from the UPPS‐P impulsivity scale is related to dopamine D2/3 receptor availability in GD (Clark *et al.* 2012) and also to MOR availability (Mick *et al.* 2015). As the main inhibitory neurotransmitter in the human brain, the role of GABAergic functioning in impulsivity is attracting attention with growing preclinical evidence supporting its involvement (Hayes *et al.* 2014). For instance, a GABA agonist and antagonist in the prefrontal cortex of rats increased and reduced, respectively, impulsive responses in the 5‐choice serial reaction time task (Murphy *et al.* 2012; Paine *et al.* 2011). GABA_A_ receptor binding is lower in the anterior cingulate cortex of high‐impulsive rats compared with low‐impulsive rats and is inversely correlated with impulsive responding (Jupp *et al.* 2013). Caprioli *et al.* reported increased impulsive behaviour in rats following a reduction in glutamate decarboxylase 65/67, an enzyme responsible for the decarboxylation of glutamate to GABA, gene expression in the NAc core (Caprioli *et al.* 2014).

In this study, we therefore used [^11^C]Ro15‐4513 PET to measure GABA_A_ receptor availability in GD and its relationship with impulsivity. Based on our previous studies showing lower [^11^C]Ro15‐4513 binding in the NAc in both alcohol and in opiate addiction, we hypothesized that GD would similarly be associated with lower levels in the NAc. In addition, based on the preclinical evidence, we hypothesized that impulsivity, specifically the NU trait, in GD would be associated with higher [^11^C]Ro15‐4513 binding; we have shown that [^11^C]Ro15‐4513 is sensitive to GABA and low levels of GABA are associated with higher [^11^C]Ro15‐4513 binding (Stokes *et al.* 2014).

## Materials and Methods

### Participants

Study participants comprised of 15 male treatment‐seeking individuals with GD (DSM‐IV) (34.8 ± 7.5 years, four current and two ex‐smokers (mean ± standard deviation (SD)) and 19 male age‐matched HV (31.7 ± 7.5 years, six current and three ex‐smokers). Individuals with GD were recruited from the National Problem Gambling Clinic, Central North West London NHS Foundation Trust, UK, and participated in the study either before (*n* = 7) or during (*n* = 8) their 8‐week programme of cognitive–behavioural therapy. HV were recruited by advertisements in daily newspapers or from our database. Written informed consent was obtained before participation in the study, which was approved by West London Research Ethics Committee and the Administration of Radioactive Substances Advisory Committee, UK.

Following a detailed telephone eligibility interview, participants attended a screening visit to assess their current and previous medical and psychiatric health. Disordered gambling was assessed with the Massachusetts Gambling Screen (6.7 ± 1.9; a score of 5 or more indicates GD) and the Problem Gambling Severity Index (16.9 ± 3.5; a score of 8 or above indicates disordered gambling). All individuals with GD had a recent history of active gambling with length of abstinence ranging from 3 to 158 days (63 ± 49 days). HV had no current or past history of psychiatric disorders (ICD‐10 or DSM‐IV Axis I diagnostic criteria). As depression and anxiety disorders are common comorbidities in GD, a history of these was permitted, but not a current depression or anxiety disorder. Past recreational drug use was allowed (>10 times in lifetime but never met criteria for abuse or dependence: seven HV: five cannabis, one cannabis and stimulants and one cannabis, hallucinogens and sedatives; four GD: two cannabis and two cannabis, stimulants and cocaine), but abstinence from illicit drugs 2 weeks before and during study participation was required. Current or past diagnosis of substance dependence, except nicotine, was an exclusion criterion for both groups. Participants were asked not to drink more than 21 UK units of alcohol (166 g) per week 2 weeks before and during the study. Urine drug screens (for cocaine, amphetamine, methamphetamine, morphine, methadone, benzodiazepines and tetrahydrocannabinol) and alcohol breath analyses were performed at screening and PET scanning days. Cigarette smoking was not allowed 1 hour prior to each magnetic resonance (MR) or PET scan. All participants had laboratory (haematology, clinical chemistry and clotting parameters) within normal range. None of the participants took regular medication.

Depressive symptoms were measured by using the Beck Depression Inventory and anxiety with the Spielberger State/Trait Inventory. Tobacco use was evaluated in the smokers with the Fagerstrom Test for Nicotine Dependence. To assess impulsivity, participants completed the UPPS‐P Impulsive Behavior Scale (Cyders *et al.* 2007), a 59 item self‐report questionnaire with five subscales: Negative Urgency (NU), Positive Urgency, Lack of Planning (LoP), Lack of Perseverance and Sensation Seeking.

### MR and PET imaging

On the screening day, a structural magnetic resonance imaging (MRI) was performed on a 3 T MR scanner (Magnetom Trio Syngo MR B13 Siemens 3 T; Siemens AG, Medical Solutions), including a volumetric T1‐weighted magnetization‐prepared rapid acquisition gradient‐echo sequence. All structural images were reviewed by an experienced neuroradiologist for unexpected findings of clinical significance and structural variation that might affect quantitative image analysis, and none were observed. Participants also completed a functional MRI battery (Paterson *et al.* 2015) and performed a neurocognitive assessment whose results will be described elsewhere.

For the [^11^C]Ro15‐4513 PET scan, we followed our previous protocol (Lingford‐Hughes *et al.* 2012a). Briefly, each participant underwent a 90‐minute [^11^C]Ro15‐4513 PET acquisition. PET data were reconstructed into 23 frames (4 × 15, 4 × 60, 2 × 150, 10 × 300 and 3 × 600 seconds) and corrected for head motion by using normalized mutual information rigid‐body registration of each frame realignment to frame 16. A hierarchical probabilistic atlas of 119 regions (Tziortzi *et al.* 2014) was non‐rigidly registered to PET space by using reverse deformation parameters derived from the normalization of the coregistered structural MRI to a standard template. Atlas fits were checked visually, and PET dynamic data were sampled in each region of interest (ROI) to generate time‐activity curves, combining left and right ROIs to generate single time‐activity curves where laterality was not hypothesized, i.e. in amygdala and orbitofrontal cortex (OFC). A parent plasma input function was derived by calibrating the continuous online blood counts to discrete samples and correcting for plasma fraction and radiolabelled metabolites collected at intervals throughout the scan. The [^11^C]Ro15‐4513 *V_T_* in each ROI could then be quantified by using a 2‐tissue‐compartmental model. This was shown, by parsimony criteria, to describe appropriately the ligand kinetics in the range of ROIs considered (Myers *et al.* 2016a). In this dataset, spectral analysis to partition this signal was not applied since we have shown the total *V_T_* to represent robustly α5 subtype binding (Myers *et al.* 2016a; Myers *et al.* 2012). All image and kinetic analysis procedures were carried out by using MIAKAT^TM^ (Imanova Ltd, UK).

### Statistical analysis

Demographic differences between groups, and injected mass/activity, were analysed by using independent‐samples *t*‐tests (two‐tailed). An omnibus mixed‐model ANCOVA tested [^11^C]Ro15‐4513 *V_T_* as a function of ROI (four levels) and Group (HV and GD) including age and smoking as covariates. Results are presented as mean + SD. Correlations between *V_T_* values and clinical variables were performed by using Spearman's rank correlation coefficients.

Based upon our previous findings in alcohol and opiate dependence for the [^11^C]Ro15‐4513 PET analysis, we selected right hippocampus and NAc as a priori ROIs, and we chose two additional a priori ROIs based upon their established role in impulse control: OFC and amygdala (Goldstein and Volkow 2002; Jentsch and Taylor 1999; Ko *et al.* 2015; Nikolova *et al.* 2016). As previous work (Clark *et al.* 2012; Michalczuk *et al.* 2011) has shown that NU, as a mood‐related subgroup of impulsivity, is most strongly associated with GD, we chose to test for correlations between UPPS‐P NU and a priori selected brain regions. Correlations were considered significant at *p* < 0.05, and for the group comparisons, if ROIs are to be treated as independent, multiple comparison correction adjusts the significance value to *p* < 0.0125.

## Results

### Participants' characteristics

Demographic, clinical, and impulsivity data are summarized in Table 1. There were no differences in age, intelligence quotient, smoking status or amount of alcohol consumed per week. Individuals with GD had greater levels of depression (Beck Depression Inventory: *p* = 0.013) and anxiety [Spielberger Trait Anxiety Inventory (STAI): *p* = 0.001; Spielberger State Anxiety Inventory (SSAI): *p* = 0.014] and Alcohol Use Disorders Identification Test (AUDIT) score (*p* = 0.037) than HV, although none of the participants reached a clinically significant threshold in either group.

**Table 1:** Participants' characteristics, clinical and impulsivity measures, mean ± SD.

	HV *n* = 19	GD *n* = 15	Significance (two‐tailed)
Age	31.7 ± 7.5	34.8 ± 7.5	0.245
IQ	114.0 ± 13.5	116.5 ± 12.2	0.502
CPGI	0.2 ± 0.5	16.9 ± 3.5	0.001*
Gambling abstinence (days)	‐	63.3 ± 50.0	‐
AUDIT	4.2 ± 2.8	6.5 ± 3.5	0.037*
Alcohol (units/ week)	9.0 ± 8.0	12.5 ± 7.4	0.182
Current smoking status (smoker/ex‐smoker)	6/3	4/2	0.957
FTND	1.0 ± 1.8	2.4 ± 2.3	0.266
Cigarettes (per day)	6.0 ± 5.8	5.0 ± 7.1	0.793
BDI	1.3 ± 2.8	4.9 ± 5.0	0.013*
STAI	31.0 ± 9.0	42.5 ± 9.9	0.001*
SSAI	26.0 ± 5.1	36.3 ± 13.8	0.014*
UPPS‐P NU	21.7 ± 5.8	30.7 ± 6.2	0.001*
UPPS‐P PU	20.7 ± 6.9	25.0 ± 8.0	0.130
UPPS‐P LoP	20.2 ± 4.7	24.3 ± 5.6	0.028*
UPPS‐P LoPe	18.2 ± 4.4	21.1 ± 4.8	0.076
UPPS‐P SS	34.3 ± 8.1	33.3 ± 7.7	0.722

GD, individuals with gambling disorder; HV, healthy volunteers; AUDIT, Alcohol Use Disorders Identification Test; BDI, Beck Depression Inventory; CPGI, Canadian Problem Gambling Inventory; FTND, Fagerstrom Test for Nicotine Dependence; GUQ, Gambling Urges Questionnaire; NART, National Adult Reading Test; SSAI, Spielberger State Anxiety Inventory; STAI, Spielberger Trait Anxiety Inventory.

* Significant difference between groups.

### PET measures

There were no significant differences (*p* > 0.05) between the groups for injected [^11^C]Ro15‐4513 mass HV: 2.98 ± 1.06 µg versus GD: 3.19 ± 1.24 µg nor for injected radioactivity HV: 352 ± 77 MBq versus GD 356 ± 90 MBq.

The omnibus ANCOVA including age and smoking (Fagerstrom Test for Nicotine Dependence) for the a priori regions revealed a significant main effect of Group (*F*_1,28_ = 6.1, *p* = 0.020), with greater mean *V_T_* in individuals with GD compared with HV. This was driven by significant group differences in the [^11^C]Ro15‐4513 total distribution volume (*V_T_*) in the right hippocampus [*t*(32) = 2.7; *p* = 0.011], which survives Bonferroni correction for multiple comparisons (Table 2). We did not show any significant difference in the NAc, OFC or amygdala (*p* > 0.05). There was also a significant main effect of smoking (*F*_1,28_ = 4.8, *p* = 0.037), such that Fagerstrom scores correlated negatively with mean *V_T_* across the four ROIs (*r_s_* = −0.36; *p* = 0.041). In both groups, there was no significant correlation between Alcohol Use Disorders Identification Test scores and [^11^C]Ro15‐4513 *V_T_*. There was also no significant correlation between ‘length of abstinence’ and [^11^C]Ro15‐4513 *V_T_*. There was no significant difference between those that had started and not started their cognitive–behavioural treatment (*p* > 0.05) in [^11^C]Ro15‐4513 *V_T_* in any of the a priori ROIs.

**Table 2:** Comparison between groups of [^11^C]Ro15‐4513 *V_T_* in a priori defined brain regions, mean ± SD.

	HV *n* = 19			GD *n* = 15			
	Mean		SD	Mean		SD	Significance (two‐tailed)
Orbitofrontal cortex	6.99	±	0.75	7.18	±	0.87	0.563
Amygdala	7.44	±	0.65	7.97	±	0.87	0.058
Hippocampus R	7.44	±	0.72	8.13	±	0.74	0.011*
Nucleus accumbens	9.76	±	0.72	9.95	±	1.04	0.544

GD, individuals with gambling disorder; HV, healthy volunteers; L, left hemisphere; R, right hemisphere. HV: Amygdala *n* = 18; GD: N. Accumbens *n* = 14.

* Significant at *p* < 0.05.

### Relationship between PET measures, impulsivity and anxiety

**Figure 1:**
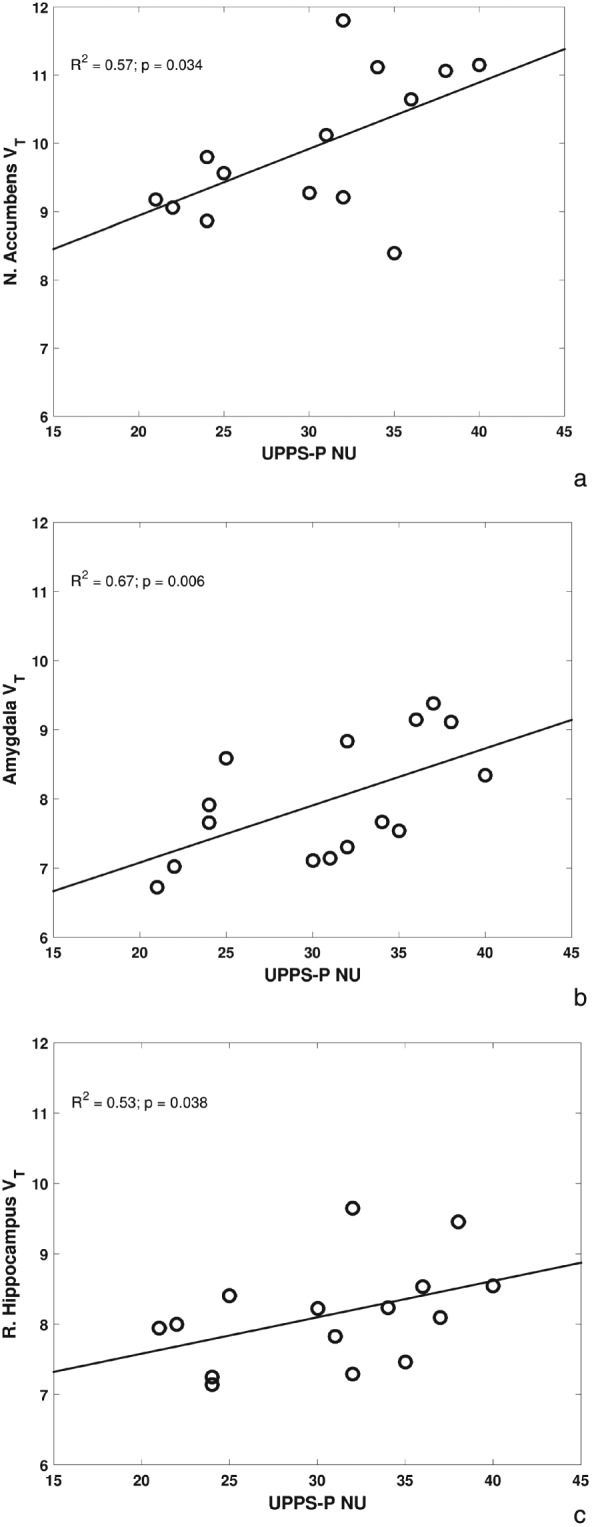
Significantly positive correlations between Negative Urgency impulsivity and [^11^C]Ro15‐4513 *V_T_* in individuals with gambling disorder

Individuals with GD showed significantly higher impulsivity scores in UPPS‐P NU (*p* < 0.001) and LoP subscales (*p* = 0.028) compared with HV (Table 1). Non‐parametric correlations were run between [^11^C]Ro15‐4513 *V_T_* and UPPS‐P NU and revealed in the GD group, significant positive correlations in the amygdala (*r_s_* = 0.67; *p* = 0.006), right hippocampus (*r_s_* = 0.53; *p* = 0.038) and NAc (*r_s_* = 0.57; *p* = 0.034; Table 3) (Fig. 1). There were no significant correlations in the HV group (*p* > 0.05). The differential relationship with NU in the two groups was confirmed statistically by using a generalized linear model, which showed a significant impulsivity × group interaction in the amygdala (*p* = 0.015). These interactions were non‐significant for right hippocampus and NAc (*p* > 0.05). There were no significant correlations between [^11^C]Ro15‐4513 *V_T_* in a priori ROIs and LoP in either of the groups.

**Table 3:** Spearman's correlations between UPPS‐P NU and [^11^C]Ro15‐4513 *V_T_* in the GD and HV groups.

	HV *n* = 19		GD *n* = 15	
	Correlation coefficient	Significance (two‐tailed)	Correlation coefficient	Significance (two‐tailed)
Orbitofrontal cortex	0.08	0.731	0.51	0.052
Amygdala	0.00	0.990	0.67	0.006**
Hippocampus R	−0.10	0.680	0.53	0.043*
Nucleus accumbens	0.46	0.050	0.57	0.034*

HV, healthy volunteers; GD, individuals with gambling disorder; L, left hemisphere; R, right hemisphere. HV: Amygdala *n* = 18; GD: nucleus accumbens *n* = 14.

* Significant at *p* < 0.05.

** Significant at *p* < 0.01.

As anxiety scores were significantly different in HV and individuals with GD (STAI; *p* = 0.001 and SSAI; *p* = 0.014) and anxiety is related to UPPS‐P NU, we also explored correlations between trait anxiety (STAI), state anxiety (SSAI), NU and [^11^C]Ro15‐4513 *V_T_* in the 4 ROIs. There was a significant positive correlation between STAI and [^11^C]Ro15‐4513 *V_T_* in the amygdala (*r_s_* = 0.51; *p* = 0.05) in the GD group, which was the only one that survived Bonferroni correction. After controlling for anxiety, the association between UPPS‐P NU and the amygdala [^11^C]Ro15‐4513 *V_T_* remained significant (*r_s_* = 0.63; *p* = 0.016). There were no significant correlations with trait anxiety in the HV group, and state anxiety was not significantly correlated with [^11^C]Ro15‐4513 *V_T_* in either group.

## Discussion

We report here the first study investigating GABA_A_ receptor availability in GD and show significantly greater [^11^C]Ro15‐4513 *V_T_* in the right hippocampus of individuals with GD and a positive relationship between [^11^C]Ro15‐4513 *V_T_* and impulsivity. Notably, our finding of higher [^11^C]Ro15‐4513 *V_T_* is in contrast to our previous finding of lower levels of [^11^C]Ro15‐4513 binding in the NAc in alcohol and in opiate dependence and lower levels in the hippocampus in alcohol dependence (Lingford‐Hughes *et al.* 2016; Lingford‐Hughes *et al.* 2012a). Higher [^11^C]Ro15‐4513 binding reflects greater GABA_A_ receptor availability, which may be due to increased receptor expression or, as a consequence of Ro15‐4513 being an inverse agonist, lower endogenous GABA levels (Stokes *et al.* 2014). We are unable to separate these two mechanisms within our study. Nevertheless, our [^11^C]Ro15‐4513 PET studies show clear differences in GABA_A_ receptor availability between substance addictions and a behavioural addiction with lower levels of [^11^C]Ro15‐4513 binding in both alcohol and opiate addiction not apparent in GD (Lingford‐Hughes *et al.* 2016; Lingford‐Hughes *et al.* 2012a).

In our studies in substance dependence, significantly lower [^11^C]Ro15‐4513 *V_T_* was found in the hippocampus in alcohol dependence but not in opiate dependence. In these studies, we used spectral analysis (Myers *et al.* 2012) to show that these findings in alcohol dependence were driven by the α5 subtype rather than α1; this is consistent with the high level of α5 subtype in the hippocampus and selectivity of [^11^C]Ro15‐4513 PET (Lingford‐Hughes *et al.* 2016). In the current study in GD, [^11^C]Ro15‐4513 *V_T_* in each ROI was quantified by using a 2‐tissue‐compartmental model, which we have shown to be suitable to describe the α5‐subtype‐specific signal of [^11^C]Ro15‐4513 in most ROIs (Myers *et al.* 2016a) though the contribution of other subtypes in regions with low GABA_A_ α5 density, such as the cerebellum, should also be considered.

We have previously reported higher [^11^C]Ro15‐4513 *V_T_* in various brain regions including the hippocampus, in HV smokers, which was particularly evident in ex‐smokers with levels in current smokers similar to non‐smokers (Stokes *et al.* 2013). However, these individuals were not heavy smokers, and in the current study, the amount of smoking was even lower so the majority were not dependent smokers. Given there were only a few current smokers, we are unable to explore further the impact of smoking on [^11^C]Ro15‐4513 *V_T_* binding in GD.

Consistent with previous work, we found higher impulsivity scores in individuals with GD compared with HV (Clark *et al.* 2012; Michalczuk *et al.* 2011). Preclinical studies suggest that reductions in or lower levels of GABA are associated with greater impulsivity (Boy *et al.* 2011; Murphy *et al.* 2012; Paine *et al.* 2011). We have previously shown that [^11^C]Ro15‐4513 is sensitive to GABA levels, and since it is an inverse benzodiazepine agonist, lower levels of GABA would be associated with higher [^11^C]Ro15‐4513 binding. Consistent with our hypothesis that there would be a positive relationship between impulsivity and [^11^C]Ro15‐4513 *V_T_*, we found such a relationship in subcortical limbic regions in GD but not in HV. The relationship between [^11^C]Ro15‐4513 *V_T_* and ‘NU’ construct of impulsivity adds further support to the idea that mood‐related impulsivity is especially relevant to disordered gambling (Clark *et al.* 2012; Nussbaum *et al.* 2011). It is notable that we have found NU to be associated with our PET studies of dopamine D2, MOR and now GABA‐A receptor in those with GD but not healthy controls suggesting that dysregulation in these systems has particular relevance to impulsivity in gambling (Clark *et al.* 2012; Mick *et al.* 2015). Given the association with NU, further exploration revealed a positive relationship between trait anxiety and [^11^C]Ro15‐4513 *V_T_* in the amygdala in the GD group only. Notably, the positive correlation with [^11^C]Ro15‐4513 *V_T_* and NU in the amygdala remained significant after controlling for anxiety. The amygdala is a key region involved in a number of processes such as emotional processing, fear, reward valence and neuropsychiatric disorders including addiction and anxiety (Elman *et al.* 2012; Janak and Tye 2015; van Holst *et al.* 2012). However, evidence from neuroimaging studies using non‐specific benzodiazepine radiotracers [^11^C]flumazenil PET or [^123^I]iomazenil SPET about GABA_A_ receptor availability within the amygdala in relation to these processes is limited and inconsistent. The most evidence relates to anxiety where no relationship or negative correlations have been reported in HV and both positive and negative correlations in patients with anxiety disorders (Abadie *et al.* 1999; Esterlis *et al.* 2009; Hasler *et al.* 2008; Lingford‐Hughes *et al.* 1998).

Our finding of an association between impulsivity and GABA_A_ receptors *in vivo* in individuals with GD is consistent with evidence from pharmacological challenges with GABA modulating drugs such as benzodiazepines, which suggest that increased GABAergic neurotransmission is associated with impulsivity (Hayes *et al.* 2014). Other studies in man have explored the relationship between GABA levels assessed with magnetic resonance spectroscopy. Whilst GABA measured this way is predominantly metabolic in origin (Myers *et al.* 2014; Myers *et al.* 2016b), it is interesting that a lowered magnetic resonance spectroscopy GABA signal ([GABA+]) in HV has in some studies been associated with higher levels of impulsivity. For instance, it has been shown that [GABA+] levels in dorsolateral prefrontal regions negatively correlate with urgency impulsivity (Boy *et al.* 2011); low [GABA+] in perigenual anterior cingulate cortex was associated with greater delay aversion on Cambridge gambling task (Fujihara *et al.* 2015) and higher scores on self‐report Barratt Impulsiveness Scale‐11 in one study (Silveri *et al.* 2013) but not another (Janes *et al.* 2013). Lower [GABA+] in the striatum is associated with poorer impulse control in Go‐NoGo task (Quetscher *et al.* 2015). Such relationships add to the notion that modulating GABA is a potential target for treatment of GD.

We acknowledge that the number of patients included in this study is small. Based on our previous [^11^C]Ro15‐4513 studies in alcohol and opiate dependent participants, we expected to find lower *V_T_* in the NAc. A post hoc power calculation revealed that we would need more than 150 additional [^11^C]Ro15‐4513 PET scans in order to be able to detect a statistically significant group difference in the NAc. The power calculation was performed by using the mean and SD data collected in our study, with alpha = 0.05, to show a difference with 80 percent power. This number is beyond what is ethically acceptable and feasible for a PET study, and we therefore did not continue scanning.

In summary, we provide here the first evidence of GABA_A_ dysregulation in individuals with GD with increased [^11^C]Ro15‐4513 *V_T_* in the right hippocampus. This is different to substance dependence where we have shown reduced [^11^C]Ro15‐4513 *V_T_* in the NAc (Lingford‐Hughes *et al.* 2016; Lingford‐Hughes *et al.* 2012a). As described, GD was categorized in DSM‐IV as an impulse control disorder but based on shared clinical and aetiological features is now classified as a behavioural addiction in DSM‐5. The current study adds to our and other previous studies failing to show similar findings in dopamine D2/3 receptor availability or mu opiate receptor availability between GD and substance dependence (Clark *et al.* 2012; Mick *et al.* 2015). Thus, our data suggest that the view that, as a behavioural addiction, the neuropharmacology of GD would be similar to substance addiction is now less certain. However, these differences may reflect the impact of the chronic excessive drug/alcohol exposure on the brain as opposed to the underlying addictive processes themselves. On the other hand, our PET studies and those of others all report a relationship between impulsivity in GD with dopamine D2/3, with MOR and with GABA_A_ receptor availability but not control groups. These associations suggest that pharmacological modulation may result in reduced impulsivity as an approach to treatment. Future studies are necessary to further investigate the neurobiology of this behavioural addiction in order to clearly address similarities and distinctions given the critical role for GABAergic function in addictive processes and its role in neural regulation. The evidence, albeit limited, and our data suggest that modulation of the GABA receptor system is a potential target for the treatment of GD.
